# Supplementary material for: Critical Drug Loss Induced by Silicone and Polyurethane Implantable Catheters in a Simulated Infusion Setup with Three Model Drugs
Source: Pharmaceutics. 2021 Oct 16;13(10):1709. doi: 10.3390/pharmaceutics13101709 (PMC8539077; doi:10.3390/pharmaceutics13101709)
Supplement: Supplementary file 1 [file pharmaceutics-13-01709-s001.zip › supplementary/pharmaceutics-1389377-sm.pdf]

pharmaceutics

MDPI

Supplementary Materials: Critical drug loss induced by silicone and polyurethane implantable catheters in a simulated infusion setup with three model drugs

Nicolas Tokhadzé, Philip Chennell , Bruno Pereira , Bénédicte Mailhot-Jensen and Valérie Sautou

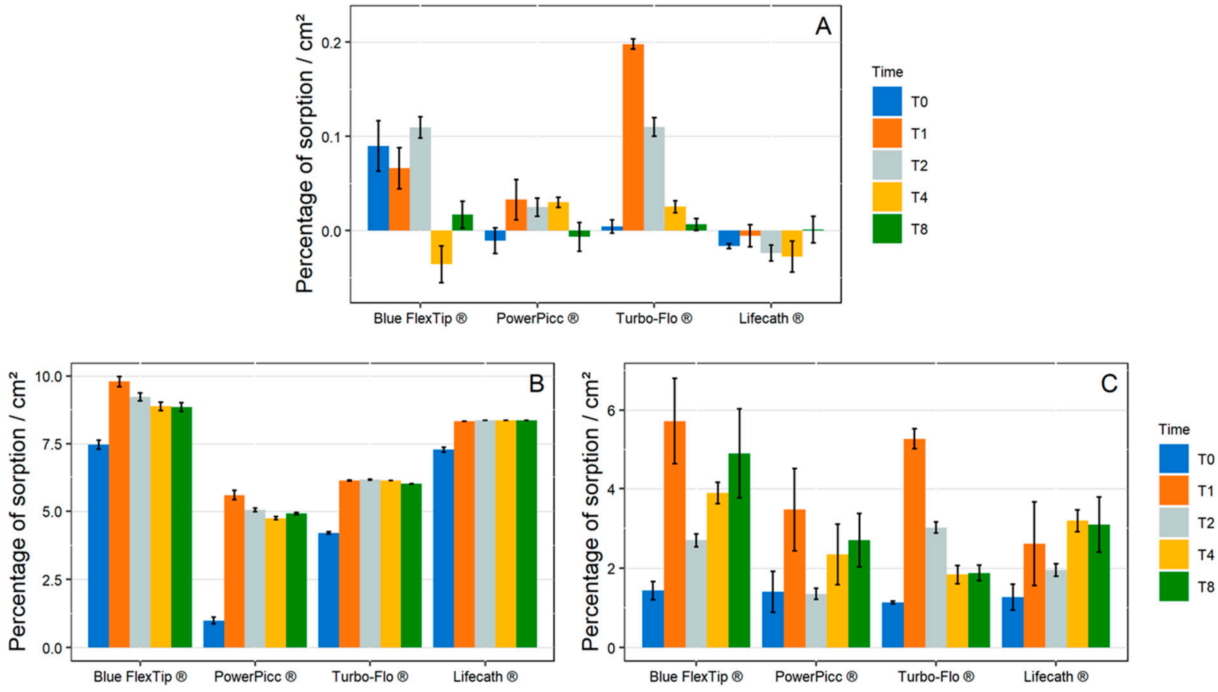

Figure S1. Percentage of sorption/cm² of paracetamol (A), diazepam (B) and insulin (C) in 1 mL/h dynamic condition with polyurethane catheters (Blue FlexTip®, PowerPicc® and Turbo-Flo®) and silicone catheters (Lifecath®). (n = 3, mean ± standard error of the mean).

Syringes

When using only an electric syringe pump, paracetamol concentrations did not differ from the initial concentrations throughout the infusion for both studied flow rates (A).

A decrease in diazepam concentrations was observed for both flow rates. However, this loss was less important with the high flow rate. At T8, a loss of 39.2 ± 2.3% was observed at 1 mL/h while it was only of 11.7 ± 1.0% at 10 mL/h. During contact with the insulin solution, a loss of around 20% was observed from T0, but the concentration then remained stable at all other analytical times for the two studied flow rates.

A complementary study was carried out to determine which parts of the syringe were involved in the observed loss of API. Syringes containing 15 or 25 mL of a diazepam or 25 mL of an insulin solution were stored upright for 96 hours in the dark in a climate chamber. The plunger stopper was either in contact with the solution or raised to avoid contact between the stopper and the drug. As shown in supplementary data (Figure B), the diazepam concentration was of 58.8 ± 0.8% of the initial concentration after 96 hours in contact with the plunger stopper with a 15 mL fill volume. The decrease in concentration was less important for the 25 mL filling volume (43.9 ± 0.5% at T96). However, when the drugs were not in contact with the plunger stopper, the diazepam concentration remained close

to the initial concentration (less than 3% variation). The changes in insulin concentration tested with or without contact with the plunger stopper were similar.

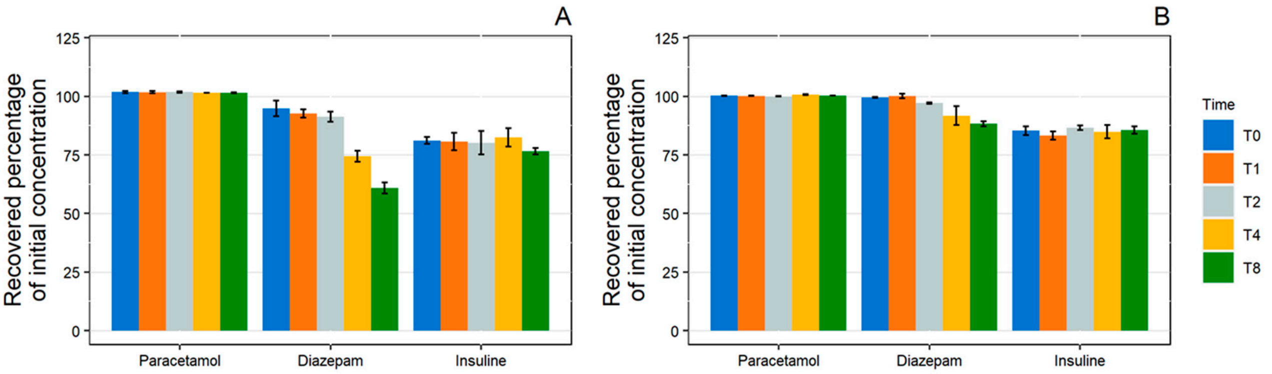

Figure S2. Evolution of the recovered percentage of initial concentration in paracetamol, diazepam and insulin in 1 mL/h dynamic condition (A) and 10 mL/h dynamic condition (B) with polypropylene syringes.

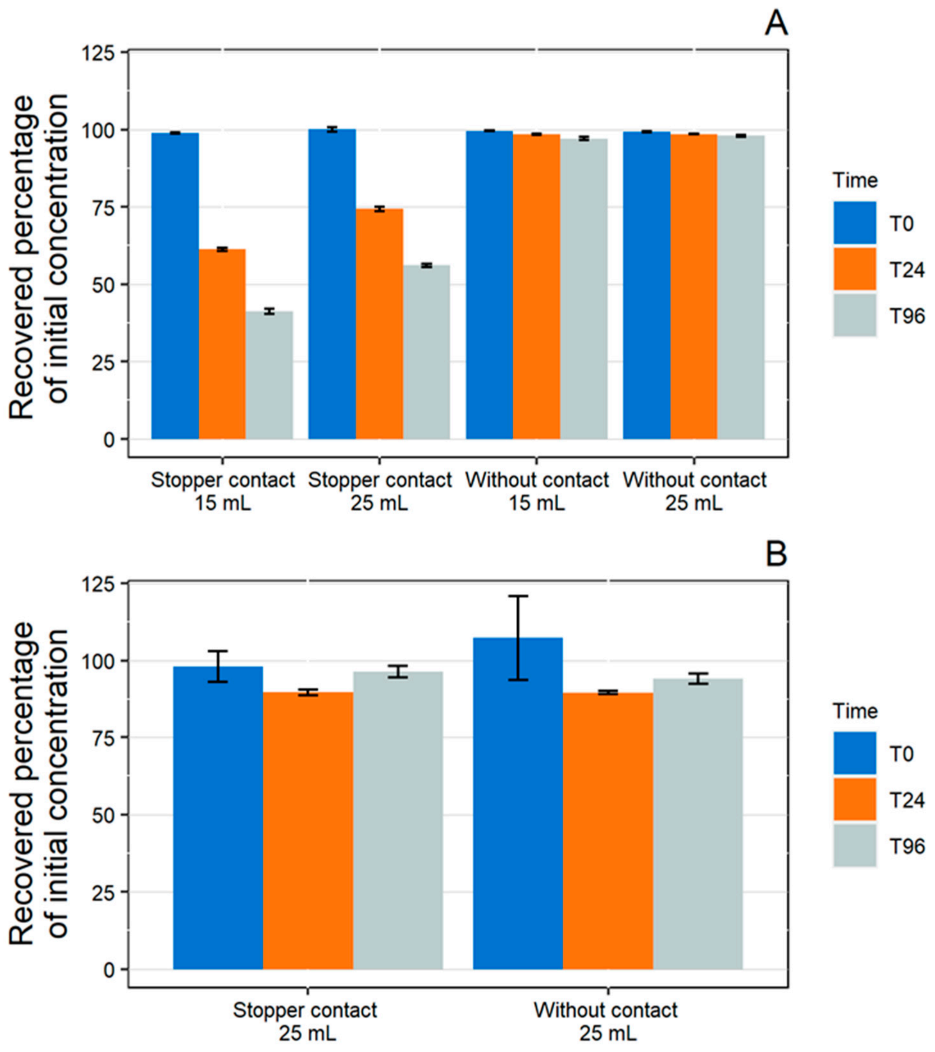

Figure S3. Evolution of the concentration of diazepam (A) and insulin (B) relative to the initial concentration after static contact with syringes (polypropylene body only or polypropylene body + polyisoprene plunger stopper) (n = 3, mean ± standard error of the mean).
